# High Sensitivity and Selectivity of AsP Sensor in Detecting SF_6_ Decomposition Gases

**DOI:** 10.1038/s41598-018-30643-y

**Published:** 2018-08-13

**Authors:** Wang Jin, Yang Guofeng, Xue Junjun, Lei Jianming, Cai Qing, Chen Dunjun, Lu Hai, Zhang Rong, Zheng Youdou

**Affiliations:** 1 Key Laboratory of Advanced Photonic and Electronic Materials, School of Electronic Science and Engineering, Nanjing University, Nanjing, 210093 China; 2 School of Science, Jiangsu Provincial Research Center of Light Industrial Optoelectronic Engineering and Technology, Jiangnan University, Wuxi, 214122 China; 3 School of Electronic Science and Engineering, Nanjing University of Posts and Telecommunications, Nanjing, 210023 China

## Abstract

The sensing properties of monolayer arsenic phosphorus (AsP) for the adsorption of SF_6_, H_2_O, O_2_, and SF_6_ decomposition gases (SO_2_ and H_2_S) are theoretically investigated by the first-principle calculations. We calculate the adsorption energy, equilibrium distance, Mulliken charge transfer, and electron localization function (ELF) to explore whether AsP is suitable for detecting SF_6_ decomposition gases. By comparing the adsorption performance of SF_6_, H_2_O, O_2_, and H_2_S gases, we have revealed that the SO_2_ gas molecules could form stable chemisorption with AsP monolayer. The results demonstrate that AsP is highly sensitive and selective to SO_2_ gas molecules with robust adsorption energy and apparent charge transfer. Furthermore, the current-voltage (*I*–*V*) curves reveal that only the adsorption of SO_2_ can largely modify the resistance of AsP. Our results show that gas sensors based on AsP monolayer could be better than that of black phosphorene (BP) to diagnose the state of online gas-insulated switchgear (GIS).

## Introduction

Sulfur hexafluoride (SF_6_) is widely used in gas-insulated switchgear (GIS) due to its excellent thermal conductivity, high dielectric strength, arc-extinguishing properties, and chemical inertness^1,2^. However, trace amounts of O_2_ and H_2_O are unavoidable impurities in GIS^3^. With time going by, the internal insulation defects and aging in GIS equipment may cause partial discharge, which will decompose SF_6_ into SO_2_, H_2_S, and other decomposition products^4,5^. These decomposition products will further accelerate insulation deterioration in GIS, and even affect the normal work of the electric equipment. Therefore, the online detection of the SF_6_ decomposition gases in GIS is essential and significant to reduce unnecessary losses caused by the breakdown of GIS equipment. The sensing methods for SF_6_ decomposition gases include gas chromatography, mass spectrometry, infrared (IR) spectroscopy, ion mobility spectrometry, and metal oxides sensors and so on^6–8^. However, these methods are not suitable for online detection because most of them require sophisticated instruments, well-trained operators, or special operating environment.

Gas sensors based on two-dimensional (2D) materials have drawn considerable attention due to their prominent advantages such as simple, cost-effective, and portable as well as high precision and sensitivity^9,10^. A lot of 2D materials, such as graphene, phosphorene, MoTe_2_ and so on, have been applied to detect the SF_6_ decomposition gases^11–13^. Arsenic phosphorus (AsP) monolayer, which is a phosphorene analogue formed from a 1:1 stoichiometric mixture of P and As. Surprisingly, the electron mobility of AsP monolayer along the armchair direction (~10000 *cm*^2^ *V*^−1^ *s*^−1^) is 1 order of magnitude larger than that of the black phosphorene (BP)^14^. It’s well known that higher carrier mobility is beneficial to gas sensor applications. More importantly, As_x_P_1−x_ (x = 0~0.83) was successfully synthesized recently by using alloying strategy^15^. It has been reported that Si-doped AsP displays an excellent sensitivity for H_2_S molecules^16^. However, there is no previous work reported whether monolayer AsP is suitable for detecting SF_6_ decomposition gases. Therefore, we firstly investigate the sensing performances of AsP for detecting the main decomposition gases of SF_6_ (SO_2_ and H_2_S) with consideration of the background gas (SF_6_, H_2_O, and O_2_) by using First-Principles.

## Results

**Figure 1:**
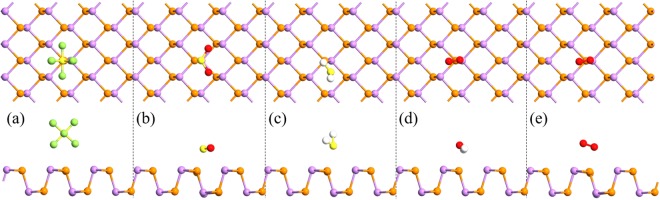
The top side views of the most stable adsorption structures of the small gas molecles: (**a**) SF_6_, (**b**) SO_2_, (**c**) H_2_S, (**d**) H_2_O and (**e**) O_2_ on monolayer AsP. (The purple and yellowish-brown balls represent As and P atoms, where yellow, green, red, and white represent S, F, O, H atoms, respectively).

The most stable configurations of the different gas molecules adsorption on AsP monolayer are illustrated in Fig. 1, and the corresponding *E*_*a*_, *d*_*0*_ and *Q* are listed in Table 1. The positive sign of *Q* means charge transfer from monolayer AsP to the adsorbates. As listed in Table 1, the equilibrium distance of SF_6_, SO_2_, H_2_S, H_2_O, and O_2_ on the AsP monolayer (3.09, 2.59, 3.07, 2.51, and 2.80 Å, respectively) are larger than P-F (1.75 Å), P-S (2.14 Å), P-H (1.43 Å), and P-O (1.74 Å) bonds^17^. The *E*_*a*_ of the most energetically favorable structures for SF_6_, SO_2_, H_2_S, H_2_O, and O_2_ molecules adsorbed on AsP are −0.480, −1.031, −0.069, −0.433, and −0.342 eV, respectively. Clearly, the adsorption energy of H_2_S on AsP is significantly smaller than the others, indicating that the AsP monolayer is not suitable for sensing this molecule. The *E*_*a*_ value of SO_2_ adsorption on AsP monolayer is also larger than that of SO_2_ adsorption on BP (−0.748 eV)^18^, indicating that a higher level sensitivity for SO_2_ detection with AsP than that with BP.

**Table 1:** The Adsorption Energy, Equilibrium Distance, and Mulliken Charge Transfer of Different Molecules Adsorbed on Arsenic Phosphorus Monolayer.

Molecule	*E*_*a*_ (eV)	*d*_*0*_ (Å)	*Q* (e)
SF_6_	−0.480	3.09 (P-F)	−0.053
SO_2_	−1.031	2.59 (P-S)	0.151
H_2_S	−0.069	3.07 (P-S)	−0.065
H_2_O	−0.433	2.51 (P-H)	0.012
O_2_	−0.342	2.80 (P-O)	0.013

**Figure 2:**
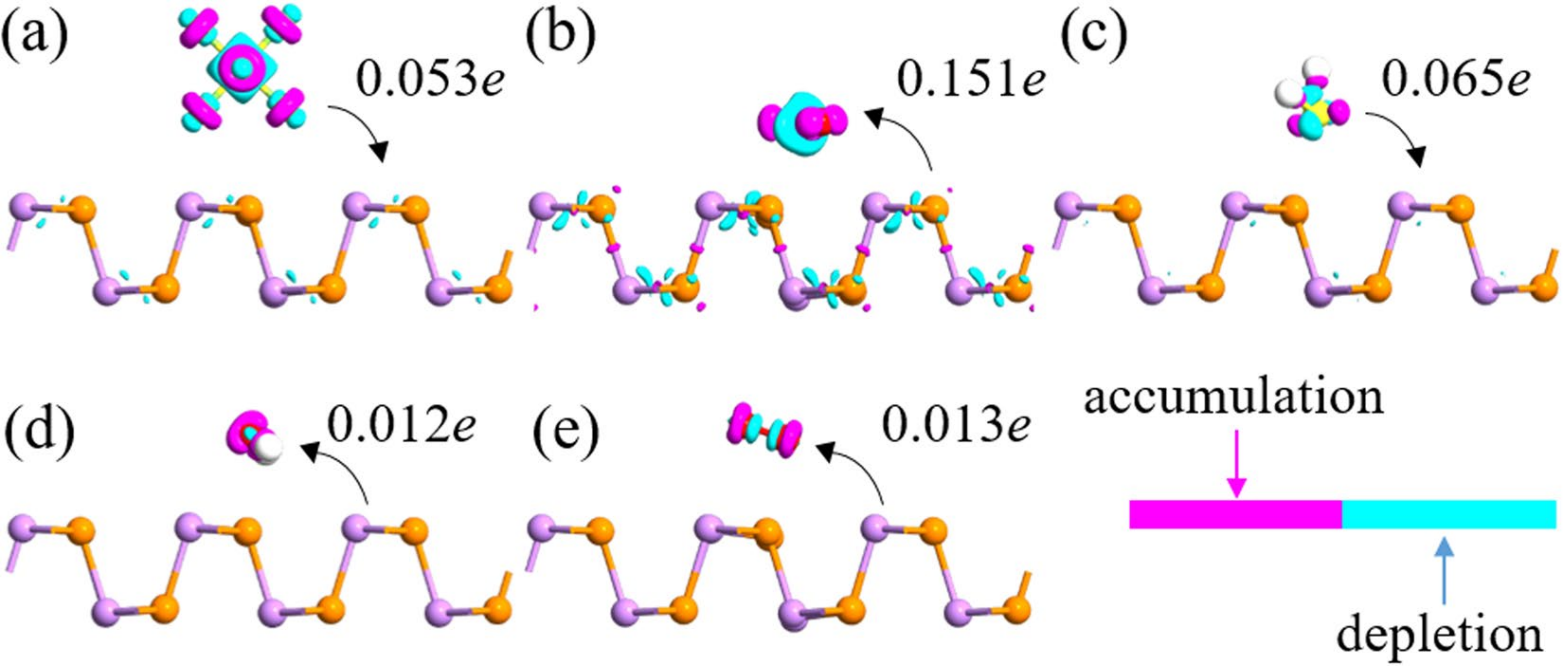
The side views of EDD calculation for (**a**) SF_6_, (**b**) SO_2_, (**c**) H_2_S, (**d**) H_2_O, and (**e**) O_2_ adsorbed on the AsP monolayer. The isovalue is 0.17 au. The cyan and purple regions indicate electron depletion and accumulation, respectively. The direction of charge transfer is shown by the arrow.

Charge transfer is another important factor to estimate the sensitivity of gas sensors. To further explore the adsorption properties between gas molecules and the AsP monolayer, the electron difference densities (EDD) are shown in Fig. 2. The Mulliken charge transfer results for SF_6_/H_2_S-AsP systems show that the charge is depleted on gas molecules and accumulated on the AsP surface, while the other three systems are exactly reversed. When SF_6_ and H_2_S molecules are adsorbed on AsP surface, they usually act as charge donors and provide 0.053 and 0.065 *e* to the AsP monolayer, respectively. H_2_O and O_2_ act as a charge acceptor and obtains 0.012 and 0.013 *e* from monolayer AsP. However, the charge transfer (*Q*) for these gas molecules is largely smaller than SO_2_-AsP system (0.151 *e* transfer from AsP to SO_2_ molecule). When we look into the EDD of SO_2_-AsP system, a much more significant charge transfer is observed. These results indicate that the electrostatic interactions between SF_6_/H_2_S/H_2_O/O_2_-AsP systems are obviously weaker than SO_2_-AsP system. Thus, the AsP monolayer is not suitable for detecting these four molecules.

**Figure 3:**
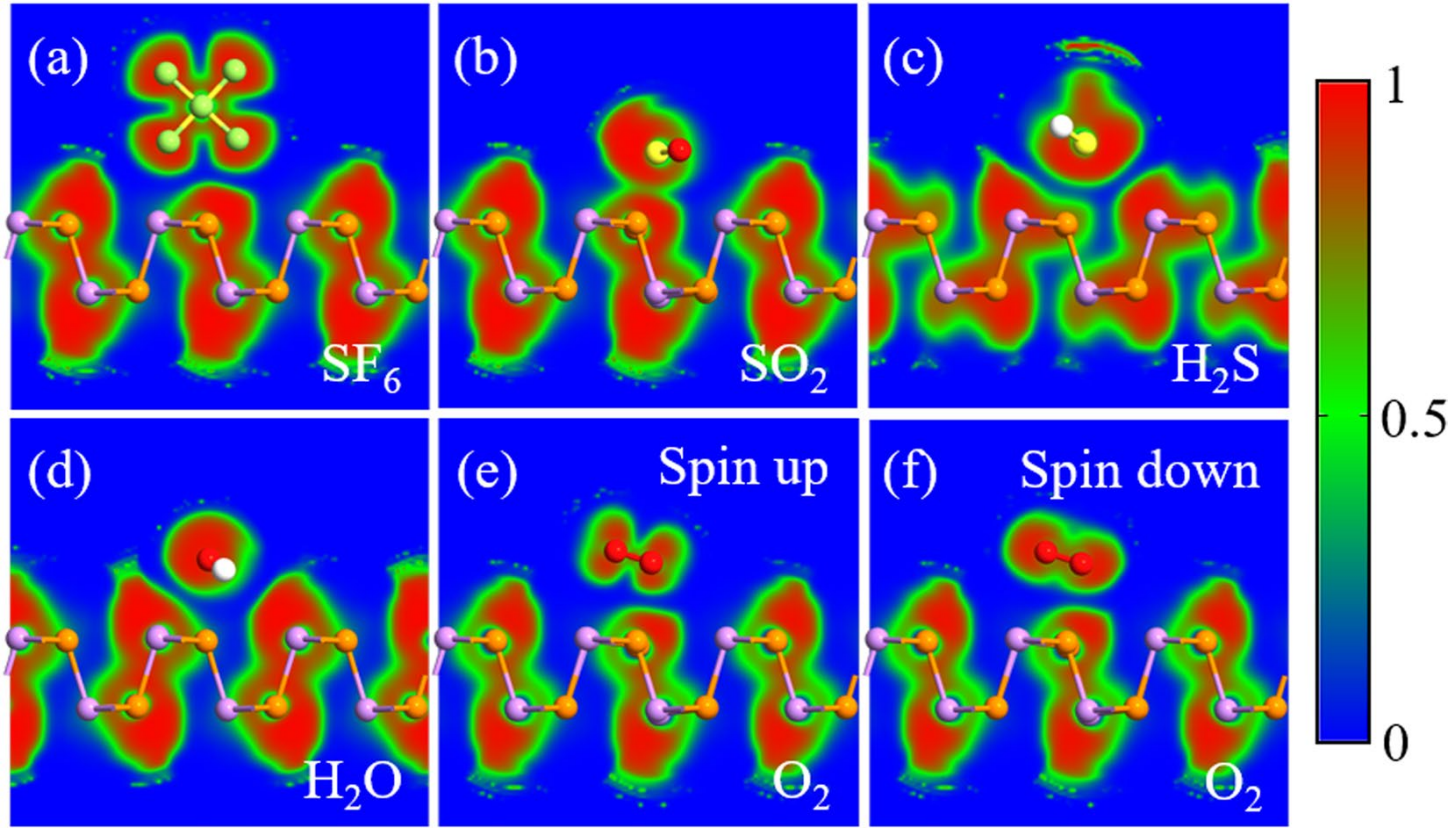
The side views of ELF calculation for (**a**) SF_6_, (**b**) SO_2_, (**c**) H_2_S, (**d**) H_2_O, and (**e**) O_2_ spin up and (**f**) O_2_ spin down adsorbed on the AsP monolayer.

**Figure 4:**
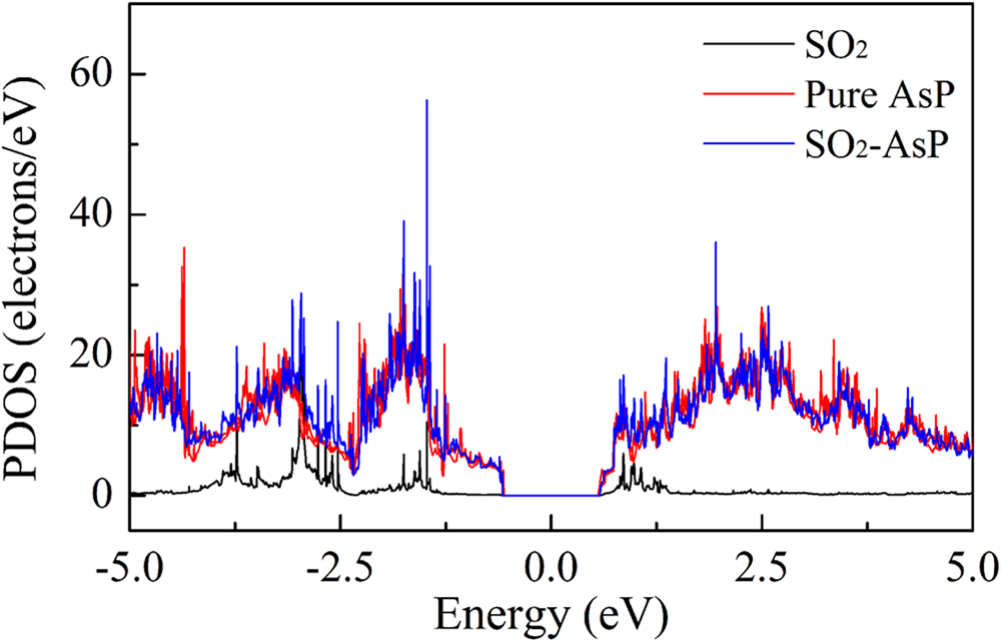
The total DOS of AsP with and without SO_2_ adsorption and the PDOS of the SO_2_ on the pure phosphorene.

To further obtain insight into the charge redistribution of the adsorption system, we plot the electron localization function (ELF) slices in Fig. 3. There is no obvious electron localization overlap between SF_6_, H_2_S, H_2_O, and O_2_ gas molecules and the AsP monolayer, which means the physisorption feature for these molecules adsorbed on AsP monolayer. The ELF of the SF_6_-, H_2_S-, H_2_O-, and O_2_-AsP systems do not have electron sharing area between gas molecules and the AsP monolayer, and thus the AsP monolayer is not sensitive to these molecules. For the SO_2_-AsP system, the electrons are slightly shared between SO_2_ molecule and AsP monolayer, revealing that the surface charge of the AsP monolayer is largely redistributed after SO_2_ adsorption. This is consistent with the result of the Mulliken charge transfer. For SO_2_ adsorption, it would be more reasonable to treat it as chemisorption due to the large binding energy, electron transfer and also slightly overlapped electron distribution as shown in Fig. 3(b). To further explore the adsorption mechanisms of SO_2_ molecules adsorbed on AsP monolayer, we plot the total electronic densities of states (DOS) and projected density of states (PDOS) in Fig. 4. Obviously, the main electronic level contributions of SO_2_ to the total system localize between −4 and −1.3 eV in the valence band, 1 and 1.3 eV in the conduction band, which is away from the Fermi level. The electrons are slightly shared between AsP monolayer and SO_2_ molecule, which reveals the intensity of the interaction between the SO_2_ molecule and the AsP monolayer. These findings imply that the strong adsorption of SO_2_ on AsP monolayer is mostly due to the electron Coulomb interaction between the lonely paired electrons of SO_2_ and the empty orbital of P atom, without hybridization^19^. Thus, we can deduce that gas sensors based on AsP are sensitive and selective to SO_2_ gas in the background of the SF_6_ decomposition gases.

**Figure 5:**
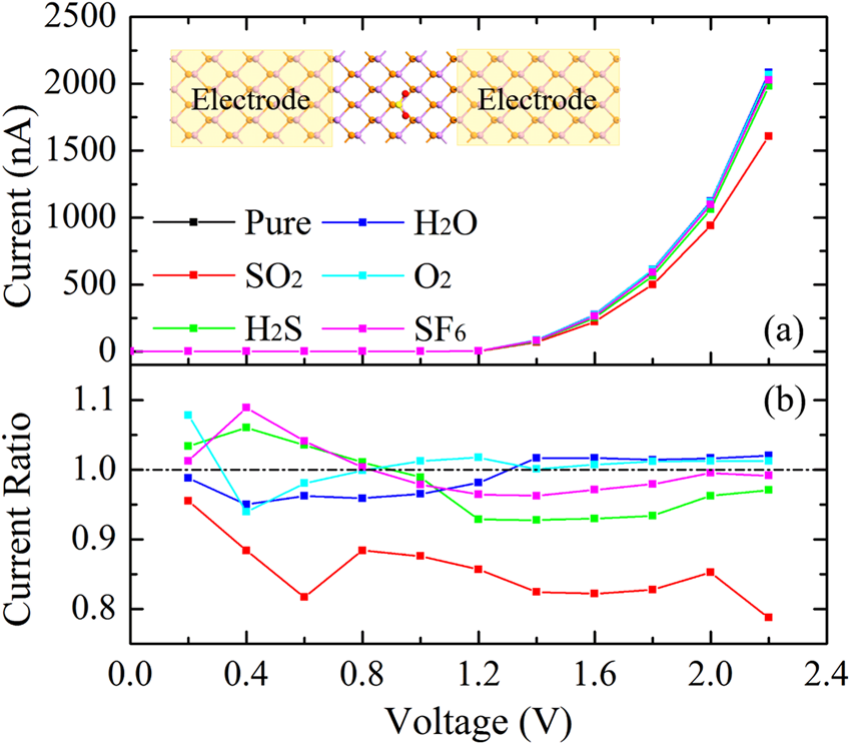
(**a**) The current-voltage (*I*–*V*) curves along the armchair direction of AsP monolayer before and after SO_2_, H_2_S, H_2_O, O_2_ and SF_6_ adsorption. The inset is the top view of the two-probe system of AsP monolayer with SO_2_ adsorption. (**b**) Current ratios of the two-probe systems with and without gas molecule adsorption.

To further verify the validity of our work, we calculate the *I*–*V* response of AsP sensor before and after the gas adsorption, as shown in Fig. 5(a). Armchair direction is chosen for transport calculation because the mobility along the armchair direction is significantly larger than the zigzag direction. There is no current (about 0.1 nA) passing through the devices when the bias voltage is smaller than 1.0 V due to the existence of band gap of pure AsP. When bias over 1.0 V, the current starts to increase dramatically. However, for the SO_2_ adsorption, with the increase of the bias voltage from 1.2 to 2.2 V, the current is clearly smaller than other cases. The reduction of current indicates the resistance of AsP is increased after the SO_2_ adsorption, which can be easily measured in the experiment. It should be emphasized that the increased resistance is caused by the larger charge transfer between the AsP monolayer and SO_2_ molecule. To gain deeper insight into the resistance change of AsP caused by the different adsorbates, we plot the current ratios before and after adsorption of gas in Fig. 5(b). It can be found that the current ratios for SO_2_ adsorption are significantly lower than that for H_2_S and SF_6_ adsorption. The value of the current reduction is about 21.3% for SO_2_ adsorption under a bias of 2.2 V, while the current reductions are 2.9% and 0.8% for H_2_S and SF_6_ adsorption respectively under the same bias. The current reduction ratio for SO_2_ adsorption is about seven times as that for H_2_S and SF_6_ adsorption, which can be easily distinguished by the magnification. The current is slightly enhanced after the H_2_O and O_2_ adsorption under a bias of 2.2 V. The resistance in AsP monolayer is highly selective and sensitive to SO_2_ in SF_6_ decomposition gases, which further demonstrates that it can be an excellent sensing material for online GIS diagnosis.

## Discussion

It was reported that BP could also be used for SO_2_ gas detection in SF_6_ decomposition gases^11^. However, the maximum current reduction is about 7% for SO_2_ adsorption, and not more than 1.5% for H_2_S and SF_6_ adsorption. For comparison purposes, we estimate the sensor response (*S*) with the formula:

$$S( \% )={\rm{\Delta }}R/R\times 100 \% ,$$

where Δ*R* is the resistance change after SO_2_ adsorption and *R* is the prior resistance of AsP monolayer. It should be emphasized that it is different to compare *S* directly in experiments because the sensitivities of 2D materials are affected by many factors, such as their thicknesses, detecting areas and so on. Nonetheless, our AsP sensor shows better response to SO_2_ than BP in the theoretical calculations, indicating that AsP monolayer could do better than BP in fabricating high sensitivity SO_2_ sensors for application in online GIS diagnosis, at least comparable to BP.

## Methods

The first principles calculations based on density functional theory (DFT) are performed using the Atomistix Tool Kit (ATK) codes at room temperature (T = 300 K)^20^. The generalized gradient approximation (GGA) with Perdew-Burke-Ernzerhof (PBE) exchange-correlation potential is adopted^21^. Fritz Haber Institute (FHI) pseudopotential using Troullier-Martins scheme with a double-ζ basis set is employed^22^. Spin polarization is only included during the calculations of the adsorption of O_2_ because it is a paramagnetic molecule. We use the Grimme’s DFT-D2 dispersion correction approach for van der Waals (vdW) corrections thanks for its higher accuracy^23^. The vacuum region is set to more than 15 Å to avoid the effect of interaction springing from the adjacent layers. A well conserved Monkhorst-Pack 8 × 8 × 1 k-point mesh is adopted for geometry optimization and electronic properties calculations with a density mesh cutoff energy of 300 Ry. Previously reported optimized lattice constants for monolayer AsP (a = 3.5 Å and b = 4.65 Å) are considered in this work^14^. We take a 3 × 3 supercell of monolayer AsP. The current-voltage (*I*–*V*) characteristics are calculated by using the nonequilibrium Green’s function (NEGF) method^24^. The k-point sampling is set to 5 × 1 × 100, and the mesh cutoff is set to 200 Ry for *I*–*V* calculation. The current of two-probe systems are calculated by the Landauer–Bütiker formula:

1 $$I={\int }_{-\infty }^{+\infty }T(E,{V}_{b})[{f}_{l}(E-{u}_{l})-{f}_{r}(E-{u}_{r})]dE$$

where *T*(*E*, *V*_*b*_) is the electron transport coefficient calculated from the Green’s functions, *f* and *u* are the Fermi-Dirac distribution function and the electrochemical potential, respectively. The subscripts “*r*” and “*l*” represent the right and left electrode.

To find the most stable configurations, we consider four different sites for each gas adsorbed on monolayer AsP, which are set up at the top of upper As/P atom, the middle of As-P bond, and the center of the puckered hexagon. The moderate distance (2.5 Å) between a single molecule and monolayer AsP layer is adopted for each initial adsorption case. On the basis of the above settings, all the configurations are fully optimized and relaxed until the force and stress tolerance are mitigated to less than 0.05 eV/Å and 0.001 eV/Å^3^, respectively. To study the interactions between monolayer AsP and targeted gas molecules, the adsorption energy (*E*_*a*_), the Mulliken charge transfer (*Q*) and the adsorption distance (*d*_*0*_) are systematically calculated. The adsorption energy is defined as:

$${E}_{a}={E}_{total}-{E}_{gas}-{E}_{AsP}$$

where *E*_*gas*_, *E*_*AsP*_, and *E*_*total*_ are the total energy of gas molecule, AsP monolayer, and gas molecule-AsP system, respectively. The adsorption distance is defined as the equilibrium’s nearest atoms between AsP monolayer and gas molecules.

## Conclusion

In conclusion, we have investigated the sensing properties of AsP monolayer for two main SF_6_ decomposition gas molecules (SO_2_, H_2_S) and three background gas molecules (SF_6_, H_2_O, and O_2_) adsorption by using the first-principles calculations. The results demonstrate that SF_6_, H_2_S, H_2_O, and O_2_ gas molecules show physical adsorption on AsP monolayer, while AsP monolayer strongly adsorb SO_2_ molecules via robust chemical bonds. It is found that the *E*_*a*_ and *Q* values of SO_2_ molecule adsorbed on AsP monolayer are obviously larger than the others, which may allow it as a desirable gas sensor for detecting SO_2_. The *I*–*V* curves demonstrate that the resistance of AsP monolayer is only largely affected by SO_2_ adsorption, indicating that the gas sensors based on AsP are highly sensitive and selective to SO_2_. Therefore, we can deduce that AsP is a promising candidate for high sensitivity and selectivity SO_2_ sensing applications in online GIS diagnosis for SF_6_ decomposition gases.
